# Radiation effects of a CT scan on chromosomal aberrations in cancer and non-cancer patients

**DOI:** 10.1093/jrr/rrag014

**Published:** 2026-05-29

**Authors:** Yuri Kawashima, Lin Shi, Wataru Fukumoto, Jiying Sun, Kimio Tanaka, Chiemi Sakai, Mari Ishida, Takafumi Ishida, Yukiko Nakano, Gloriamaris Loy-Caraos, Namkhai Bayasgalan, Suvd Bayarjargal, Yoshitaka Kamimura, Hiroshi Aikata, Shinji Yoshinaga, Kazuaki Chayama, Kazuo Awai, Satoshi Tashiro

**Affiliations:** Research Institute for Radiation Biology and Medicine, Hiroshima University, 1-2-3 Kasumi, Minami-ku, Hiroshima 734-8551, Japan; Department of Radiology, The Second Affiliated Hospital of Shandong First Medical University, Tai’an, Shandong 271000, China; Department of Diagnostic Radiology, Graduate School of Biomedical and Health Science, Hiroshima University, 1-2-3 Kasumi, Minami-ku, Hiroshima 734-8551, Japan; Department of Cellular Biology, Research Institute for Radiation Biology Medicine, Hiroshima University, 1-2-3 Kasumi, Minami-ku, Hiroshima 734-8551, Japan; Department of Cellular Biology, Research Institute for Radiation Biology Medicine, Hiroshima University, 1-2-3 Kasumi, Minami-ku, Hiroshima 734-8551, Japan; Department of Cardiovascular Physiology and Medicine, Graduate School of Biomedical & Health Sciences, Hiroshima University, 1-2-3 Kasumi, Minami-ku, Hiroshima 734-8551, Japan; Department of Cardiovascular Physiology and Medicine, Graduate School of Biomedical & Health Sciences, Hiroshima University, 1-2-3 Kasumi, Minami-ku, Hiroshima 734-8551, Japan; Department of Health and Nutrition, Faculty of Health Sciences, Hiroshima Shudo University, 1-1-1 Oozuka-Higashi, Asaminami-ku, Hiroshima 731-3195, Japan; Department of Cardiovascular Medicine, Fukushima Medical University, 1 Hikarigaoka, Fukushima 960-1295, Japan; Department of Cardiovascular Medicine, Graduate School of Biomedical & Health Sciences, Hiroshima University, 1-2-3 Kasumi, Minami-ku, Hiroshima 734-8551, Japan; Philippine Nuclear Research Institute, Department of Science and Technology, Commonwealth Avenue, Diliman, Quezon City 1101, Philippines; Department of Cellular Biology, Research Institute for Radiation Biology Medicine, Hiroshima University, 1-2-3 Kasumi, Minami-ku, Hiroshima 734-8551, Japan; Department of Cellular Biology, Research Institute for Radiation Biology Medicine, Hiroshima University, 1-2-3 Kasumi, Minami-ku, Hiroshima 734-8551, Japan; Department of Cellular Biology, Research Institute for Radiation Biology Medicine, Hiroshima University, 1-2-3 Kasumi, Minami-ku, Hiroshima 734-8551, Japan; Department of Gastroenterology and Hepatology, Hiroshima Prefectural Hospital, 1-5-54 Ujina-Kanda, Minami-ku, Hiroshima 734-8530, Japan; Department of Radiation Biophysics, Research Institute for Radiation Biology and Medicine, Hiroshima University, 1-2-3 Kasumi, Minami-ku, Hiroshima 734-8551, Japan; Hiroshima Institute of Life Sciences, 7-21 Nishi-Asahi-machi, Minami-ku, Hiroshima 734-0002, Japan; RIKEN Center for Integrative Medical Sciences, 1-7-22 Suehiro-cho, Tsurumi-ku, Yokohama 230-0045, Japan; Department of Diagnostic Radiology, Graduate School of Biomedical and Health Science, Hiroshima University, 1-2-3 Kasumi, Minami-ku, Hiroshima 734-8551, Japan; Department of Cellular Biology, Research Institute for Radiation Biology Medicine, Hiroshima University, 1-2-3 Kasumi, Minami-ku, Hiroshima 734-8551, Japan

**Keywords:** low-dose irradiation, chromosomal aberrations, PNA-FISH (fluorescence in situ hybridization method using telomere and centromere peptide nucleic acid probes), CT scans, radiation sensitivity, hepatocellular carcinoma

## Abstract

Computed tomography (CT) is indispensable in clinical practice, but the health risks of repeated low-dose radiation exposure remain unclear, especially in relation to potential differences between cancer and non-cancer patients. This study aimed to examine potential differences in biological responses to CT-associated low-dose irradiation between non-cancer patients and patients with hepatocellular carcinoma (HCC). In a prospective observational study, chromosomal aberrations (CAs) in peripheral blood lymphocytes (PBLs) were quantified by fluorescence *in situ* hybridization analysis with peptide nucleic acid probes (PNA-FISH). Data from 60 non-cancer patients obtained in our previous study were used as control, and 61 HCC patients were newly enrolled. Baseline CA frequencies were significantly higher in HCC patients (30.6 per 1000 cells) than in non-cancer patients (5.6 per 1000 cells; *P* < 0.0001). Among the 61 HCC patients, those with a history of radiotherapy (RT) exhibited higher baseline CAs (64.8 per 1000 cells) compared with those without RT (*n* = 49; 22.2 per 1000 cells). In RT-negative HCC patients, a history of TACE also correlated with increased baseline CAs (*P* < 0.05), suggesting a contribution of prior genotoxic therapies. In HCC patients without prior RT, a history of TACE also correlated with increased baseline CAs (*P* < 0.05), suggesting a contribution of prior genotoxic therapies. In addition, Notably, CT-induced CA formation was greater in HCC patients with prior RT than in patients without RT (*P* < 0.05). These results support careful assessment of cumulative medical radiation exposure in cancer patients who undergo repeated imaging and/or treatment.

## INTRODUCTION

Substantial evidence from epidemiological studies, including cohorts of the Hiroshima and Nagasaki atomic-bomb survivors, has established that moderate- to high-dose ionizing radiation increases cancer risk [1–4].

These cohorts consistently demonstrate that cancer risks rising in an approximately linear manner at doses above 100 mGy [2]. However, the health effects of low-dose radiation on cancer incidence remain uncertain. Even among well-characterized epidemiological studies of atomic bomb survivors, the effects of low-dose exposure are difficult to distinguish from background variability [2, 4–6]. Consequently, a linear non-threshold (LNT) model is currently applied in radiation protection, although further investigation is required for the practical management of low-dose radiation exposure [7, 8].

In clinical practice, diagnostic imaging, particularly computed tomography (CT), is a major source of ionizing radiation [9]. A standard CT examination typically delivers less than 100 mGy [10, 11], yet the widespread and increasing use of CT has heightened concern regarding its potential long-term effects. Several studies have suggested a possible link between CT exposure and elevated risks of leukemia or other malignancies [12–14]. More recently, in 2023, a large-scale cohort study reported increased incidences of leukemia and brain tumors in children and adolescents who underwent CT imaging [15, 16]. Accordingly, careful justification and optimization of CT are emphasized in pediatric patients and pregnant women [17, 18].

Beyond age-related sensitivity, patients with immunodeficiency show increased radiation sensitivity due to genetically impaired DNA repair mechanisms [19–22]. Our previous studies have demonstrated that even among healthy individuals, there are substantial inter-individual variation in responses to very low doses of radiation, including those delivered during CT scans [23–25]. This variability, also reported from Abe *et al.* [26, 27], reflects what we refer to as biological resilience—a composite capacity of cells to cope with radiation exposure through mechanisms such as DNA repair, cell-cycle regulation, and apoptosis. These findings indicate that radiation sensitivity is heterogeneous even within the general population. Notably, severe radiation-induced side effects are occasionally observed in cancer patients without any apparent immunodeficiency, further suggesting that individual differences in radiation-sensitivity exist even among individuals considered clinically healthy [23, 28]. Therefore, identifying individual differences in radiation sensitivity could be essential for predicting adverse effects and optimizing personalized radiotherapy strategies. Nevertheless, both the extent and underlying mechanisms of inter-individual variation in response to low-dose radiation remain poorly understood. A comprehensive evaluation of the biological effects of low-dose radiation is urgently needed to ensure the appropriate management of medical exposures.

Accumulation of genomic alterations, including chromosome aberrations (CAs), is implicated in carcinogenesis. Among CAs, dicentric and ring chromosomes are considered unstable aberrations because they are lethal to the cell and are not passed on to progeny [1–3]. Therefore, the dicentric chromosome assay (DCA) of peripheral blood lymphocytes (PBLs) is widely recognized as the ‘gold standard’ for estimating the dose of recent radiation exposure in radiation emergency medicine [29–31]. However, the association between CA formation after exposures below 100 mSv remains difficult to quantify [32, 33], partly because subtle increases are hard to detect with conventional Giemsa-stained metaphase analysis [29, 34]. Moreover, individual variability in DNA repair fidelity may further influence the degree of CA formation, even at identical radiation doses.

To address these challenges, we previously established a high-throughput fluorescence *in situ* hybridization method using telomere and centromere peptide nucleic acid probes (PNA-FISH) to quantify radiation-induced CAs in PBLs and applied it to evaluate CT effects in non-cancer patients [25, 35]. In the present study, we compared CA burden and CT-associated CA increases between non-cancer patients and hepatocellular carcinoma (HCC) patients, including subgroups defined by treatment history. Using PNA-FISH, we observed significantly higher CA numbers in HCC patients, particularly in those who had previously undergone radiotherapy (RT). Both non-cancer and HCC patients exhibited increases in CAs following CT scanning. Importantly, the magnitude of the CT-induced increase in CA was positively correlated with the dose-length product (DLP) and negatively correlated with the pre-existing CA burden, suggesting inter-individual differences in radiation sensitivity among individuals without immunodeficiency disorders.

## METHODS

This study was approved by the ethics committee of Hiroshima University (No. E579). Informed consent was obtained from all patients.

### Data collection

This study conducted with the approval of the institutional review board. Informed consent was obtained from all patients. HCC patients were enrolled from 2013 to 2015. Collected demographic and clinical data included age, gender, weight, height, body mass index (BMI), DLP, history of RT, history of transcatheter arterial chemoembolization (TACE), and the number of prior CT scans. All CA raw count data are provided in Supplementary Table S1.

### Non-cancer data source and data reuse

The non-cancer data used in this study were obtained from Shi *et al.* 2018 [25]. This dataset of 60 non-cancer individuals who underwent cardiac or hepatic CT scans, with analysis focused on the increment of CAs counting dicentric and ring chromosomes using the same PNA-FISH analysis as in the present study.

The dataset was selected because it comprised non-cancer patients and therefore served as an appropriate control group for the current analysis. All reuse of data was conducted in compliance with the same ethical guidelines and institutional regulations as those applied in this study.

### Hepatic dynamic CT

Sixty-one patients with HCC underwent hepatic dynamic CT using a 64-detector CT scanner (Light Speed VCT: GE Healthcare). Helical acquisition at the top of the liver; unenhanced and three-phase contrast medium-enhanced helical images of the entire liver were obtained. The scanning parameters were as follows: rotation time 0.45 sec., beam collimation 0.625 x 64 rows, helical pitch (beam pitch) 0.938, FOV 50 x 50 cm, voltage 120 kV, auto mA (noise index 10). The contrast dose was 600 mgI per body weight (kg). One of three contrast agents (iopamidol, iomeprol, iohexol) was selected for each patient based on body habitus and allergy history. These agents have been suggested to have comparable effects on DNA damage [36]. The contrast medium was injected into the cubital vein for 30 sec. Through the 22-G intravenous catheter using an automatic injector.

### Effective dose estimation

According to ICRP Publication No.102, a reasonable estimate of an effective dose can be achieved using the relationship: effective dose 1/4 k DLP, where k is a weighting factor (mSv·mGy^−1^·cm ^−1^) [37]. The thorax value of k is 0.014 and the abdomen value is 0.015 [38]. The effective dose of patients in the current are shown in Supplementary Table S1, and the calculation method and criteria are consistent with those in our previous study [25].

### Blood collection and human primary lymphocyte culture

Blood was collected immediately before and after each hepatic CT scan. Human PBLs were separated using Lymphoprep from 15 min after collection (AXIS-SHIELD Density Gradient Media), and cultured in RPMI-1640 medium containing 20% fetal calf serum, 2% phytohemagglutinin (Remel), and 0.05 μg/ml Colcemid (Gibco) at 37°C. After 48 hours of incubation, the hypotonic treatment and fixation of lymphocytes were performed as previously described [35]. Metaphase chromosome slides were prepared using a HANABI Metaphase Spreader (ADSTEC Corporation).

### Telomere-centromere metaphase FISH

Metaphase FISH was performed following a previously established protocol [35]. A Cy3-labeled centromere PNA probe (Cent-Cy3, Panagene) and a FAM-labeled telomere PNA probe (TelC-FAM, Panagene) were applied at the final concentration of 25 nmol/L each. After hybridization, the slides were mounted with Vectashield containing DAPI (Vector Labs).

### Image analyses

Images were acquired automatically using a CoolCube1 camera and MetaSystems imaging platform (MetaSystems Hard & Software GmbH). The chromosomal analysis of more than 1000 metaphase lymphocytes per sample was performed with the Isis imaging software (MetaSystems Hard & Software GmbH). Metaphase lymphocytes with 46 centromere signals were scored. Unstable chromosomal aberrations (CAs), particularly dicentric, tricentric, tetracentric, ring and dicentric ring chromosomes in PBLs were quantified. Abnormal chromosomes with ‘n’ centromeres were counted as ‘n-1’ dicentric chromosomes [35]. All scoring was blinded manner.

### Statistical analyses

Linear regression and statistical analyses were performed using Prism software (GraphPad Software, La Jolla, CA) and R statistical software version 4.3.2 (R Core Team, 2023). Univariate and multivariate linear regression were used to identify independent factors associated with before (baseline) and after CT incidence of dicentric and ring chromosomes as CAs among all patients. Multiple regression models were determined *a priori,* and all factors were entered into the initial model. Stepwise model selection was performed using the stepAIC function in the MASS package of R, and clinically relevant factors were incorporated to construct the optimal model. A two-sided *P* value <0.05 was considered statistically significant.

### Regression modeling of correlation with DLP and CAs

A regression model was constructed under the two assumptions below:

1) The increase of CAs after a CT scan is proportional to DLP.

Using a coefficient of variation model, which demonstrates that DIFF increases linearly with DLP, the following equation was established:


$$ {\displaystyle \begin{array}{c} DIFF=c\cdot DLP\\{}\left( DIFF: After\_ CAs- Before\_ CAs,c: coefficient\kern0.17em of\kern0.17em variation\right)\end{array}} $$

2) The regression of variation depends on baseline CA levels.

The coefficient of variation was expressed using the following linear model:


$$ {\displaystyle \begin{array}{c}c=a+b\cdot Before\_ CAs\\{}\left(a,b: regression\ coefficients\right)\end{array}} $$

Consequently, the following equation is derived:


$$ DIFF=a\cdot DLP+b\cdot \left( Before\_ CAs\cdot DLP\right) $$

## RESULTS

### Demographic and dosimetric characteristics of non-cancer and HCC patients

**Fig. 1:**
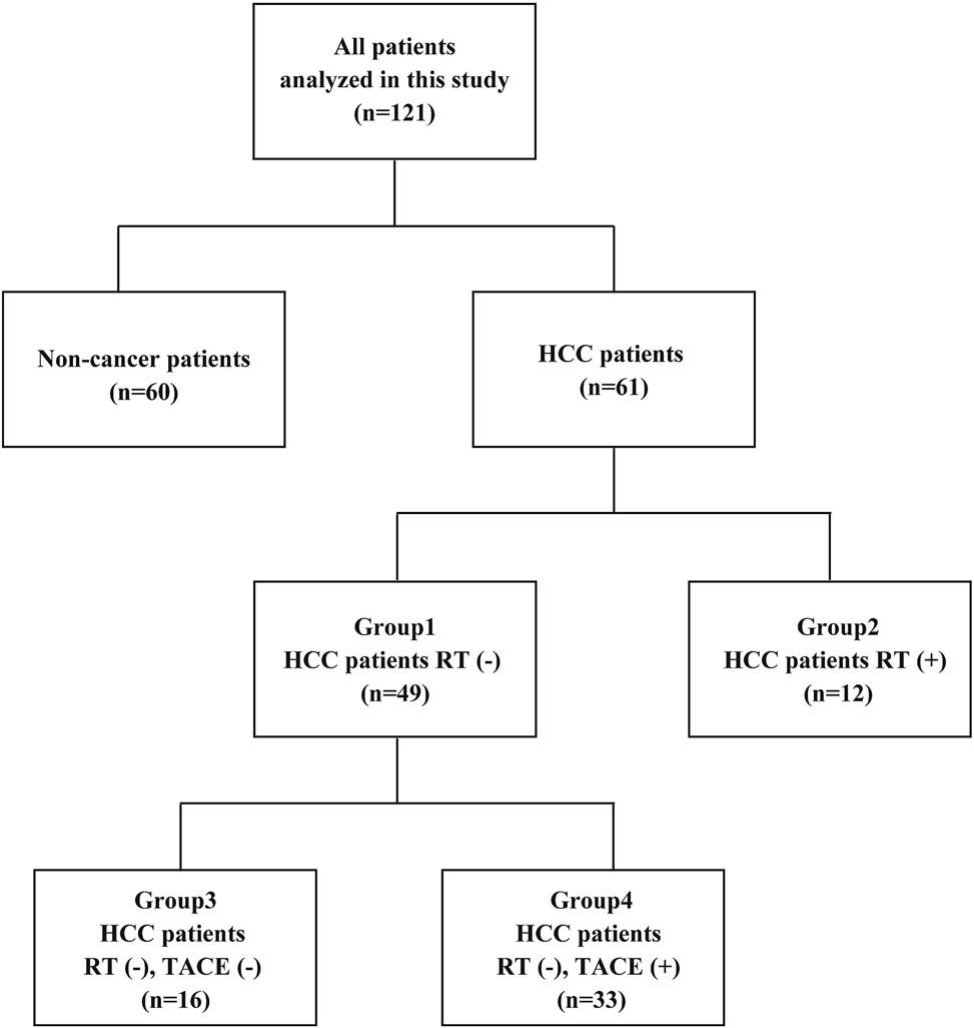
Flowchart illustrating the grouping of patients enrolled in this study. A total of 121 patients were enrolled in this study. Patients were initially divided into the HCC and non-cancer groups. The data for the non-cancer group are from the same study by Shi *et al*. 2018, as described in the Methods section. Within the HCC group, patients were further categorized based on whether they received RT or not. Group 1: HCC patients without RT. Group 2: HCC patients with RT. Group 2 patients were further divided into Groups 3 and 4 based on the history of TACE.

In our previous study, we demonstrated that the dicentric analysis using PNA-FISH of approximately 100 PBLs after *in vitro* high-dose irradiation provided higher accuracy than conventional Giemsa staining [35]. Accordingly, we applied PNA-FISH analysis to evaluate the effects of low-dose irradiation from a CT scan. That study also revealed a significant increase in CAs (*P* < 0.001) among 60 non-cancer patients following a CT scan [25]. In the present study, we newly recruited 61 patients with HCC and compared their CAs in PBLs with those of the 60 non-cancer patients from our previous study, who served as controls, to evaluate differences in responsiveness to low-dose radiation exposure (Fig. 1). We first examined demographic and dosimetric characteristics between non-cancer and HCC patients to determine any imbalances in the sampled population that could influence data. As expected, the HCC group had a significantly higher mean age, reflecting the increased incidence of HCC with advancing age. In contrast, BMI, DLP as a simple indicator of radiation exposure, and effective dose showed no significant differences between the groups (Table 1).

**Table 1:** Baseline characteristics of HCC patients

	Non-cancer patients (Shi *et al.* 2018)	HCC patients			
	All	Radiotherapy (−)	Radiotherapy (+)	All	*P*-value
n	60	49	12	61	–
Women, n (%)	15 (25.0)	9 (18.4)	5 (41.6)	14 (22.9)	–
Age (year)	60.5 ± 10.8	68.5 ± 8.9	71.0 ± 8.8	69.0 ± 8.9	^****^
Weight (kg)	66.2 ± 14.7	62.1 ± 10.3	55.7 ± 8.3	60.9 ± 10.2	^**^
Height (cm)	165.7 ± 9.8	163.5 ± 8.0	157.4 ± 6.9	162.3 ± 8.1	^*^
BMI (kg/m^2^)	23.9 ± 3.6	23.2 ± 3.2	22.4 ± 2.2	23.0 ± 3.0	^*^
DLP (mGy·cm)	1439.4 ± 676.5	1363.6 ± 660.2	1278.0 ± 696.8	1346.8 ± 662.5	ns

HCC: Hepatocellular carcinoma, DLP: dose-length product.

^****^ *P* < 0.0001, ^**^P < 0.01, ^*^P < 0.05.

The value of age, weight, height, BMI, DLP are shown mean ± SD.

### Factors affecting inter-individual differences in baseline CAs identified by regression analysis

**Fig. 2:**
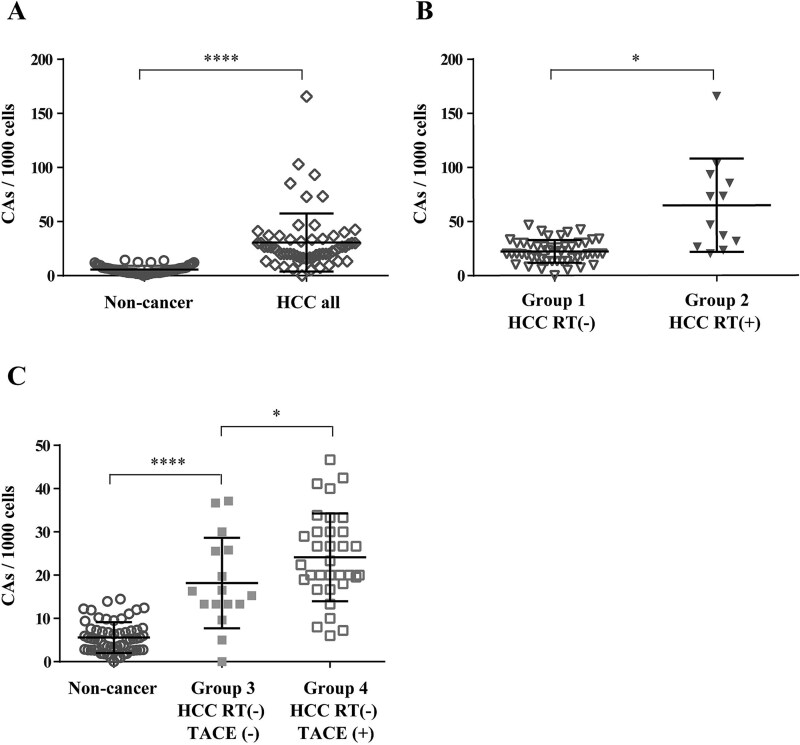
CAs in PBLs from patients before and after CT scans. (A) Baseline CAs in PBLs from non-cancer and HCC patients before CT scans. The vertical axis indicates the CAs per 1000 cells. (B) First subgroup analysis for all HCC patients with and without a history of RT. (C) Second subgroup analysis of HCC patients without RT, based on the history of TACE. Statistical significance was evaluated using the Mann–Whitney U test. Data are presented as mean ± SD. ^****^P < 0.0001, ^*^P < 0.05.

We next focused on differences in baseline CAs the two groups and quantified CAs in PBLs prior to CT scan. PNA-FISH analysis revealed that baseline CAs in non-cancer patients had a mean of 5.6 ± 3.6 CAs per 1000 cells (mean ± SD), whereas in HCC whereas those in HCC patients exhibited significantly higher baseline levels (30.6 ± 26.8 per 1000 cells, *P* < 0.0001; Fig. 2A, Supplementary Table S2). This trend was also confirmed by regression analysis among all 121 patients (Table 2). This suggests that HCC patients generally have higher baseline CAs than non-cancer patients as shown in Table 1.

**Table 2:** Univariate and multiple regression analysis for factors affecting number of CAs before CT scans among all patients

	Univariate test			Multivariate test		
Variable	Coefficient	*P*-value		Coefficient	*P*-value	
HCC	25.560	1.503 × 10^−4^	^***^	12.336	0.116	
Sex	1.342	0.843				
Age	0.408	0.173				
BMI	−0.376	0.705				
DLP	−1.641 × 10^−3^	0.726				
History of smoking	8.658	0.165		10.993	4.693 × 10^−2^	^*^
Number of CT scans	1.054	4.216 × 10^−5^	^****^	0.8199	8.086 × 10^−3^	^***^

^****^ *P* < 0.0001, ^***^P < 0.001, ^*^P < 0.05.

Beyond ‘HCC status,’ univariate regression analysis across all patients identified ‘Number of prior CT scans’ as a significant factor associated with baseline CAs (Table 2). Multivariate regression analysis, which incorporated and controlled for demographic and clinical characteristics, confirmed that the number of prior CT scans significantly influenced baseline CAs. Additionally, smoking history was identified as a significant factor. Although age is widely recognized as a determinant of increasing CA levels [39–41], our regression analysis did not identify age as a significant predictor of baseline CAs in this cohort [39–41].

Because prior chemoradiotherapy exposures in cancer patients could potentially influence baseline CAs, we performed separate regression analyses for the non-cancer and HCC group. n HCC patients, univariate regression identified ‘history of TACE,’ ‘history of RT,’ and ‘number of prior CT scans’ as significant predictors of baseline CAs. Multivariate regression analysis confirmed that a history of RT significantly influenced baseline CAs, with TACE history being marginally significant (Table 3). In contrast, non-cancer patients followed a pattern similar to the entire cohort, with number of CT scans significantly affecting baseline CAs (Table 2). Collectively, these results indicate that prior therapeutic interventions particularly RT and possibly TACE, significantly influence baseline CAs in HCC patients.

**Table 3:** Univariate and multiple regression analysis for factors affecting number of CAs before CT scans among HCC patients

	Univariate test			Multivariate test		
Variable	Coefficient	*P*-value		Coefficient	*P*-value	
History of TACE	6.753	1.247 × 10^−4^	^***^	3.813	1.400 × 10^−2^	^*^
History of RT	41.771	2.510 × 10^−7^	^****^	33.822	2.190 × 10^−5^	^****^
Sex	−4.556	0.599				
Age	0.173	0.842				
BMI	−0.546	0.352				
DLP	3.710 × 10^−3^	0.501				
History of smoking	11.500	0.129				
Number of CT scans	0.719	4.601 × 10^−2^	^*^	0.124	0.685	

^****^ *P* < 0.0001, ^***^P < 0.001, ^*^P < 0.05.

### The effects of TACE and RT for baseline CAs

Given that TACE and RT were identified as significant factors in the multivariate analysis, we next evaluated their specific effects on baseline CAs in HCC patients. HCC patients (*n* = 61) were divided into two groups based on history of RT: ‘HCC RT (-) (*n*=49)’ or ‘HCC RT (+) (*n*=12)’ (Fig. 1). Detailed RT histories for these 12 patients are provided in Supplementary Table S3. Baseline CAs were markedly increased in HCC RT (+) patients (64.8 ± 43.1 per 1000 cells) compared with those without RT (22.2 ± 10.5 per 1000 cells; Fig. 2B, Supplementary Table S4).

Because 11 of 12 HCC patients with RT also had a history of TACE, it was difficult to separate the individual contributions of RT and TACE within the RT (+) group. Thus, we instead further divided HCC RT (−) patients into two groups based on TACE history: ‘RT (–) TACE (+) (*n*=16)’ and ‘RT (–) TACE (-) (*n*=33)’ (Fig. 1). Consistent with multivariate regression analysis, TACE history had a significant influence on baseline CAs in the HCC RT (−) group. The TACE (+) group showed higher baseline CAs (24.1 ± 10.2 per 1000 cells) than the TACE (−) group (18.2 ± 10.5 per 1000 cells; Fig. 2C, Supplementary Table S5). Moreover, HCC patients who were RT (−) TACE (−) still demonstrated much higher baseline CAs than non-cancer patients (Fig. 2C). These results suggest that the history of genotoxic therapy, such as TACE, even in RT (−) HCC patients, significantly contributes to higher baseline CAs.

### Influence of prior radiotherapy on CT-induced CAs

**Fig. 3:**
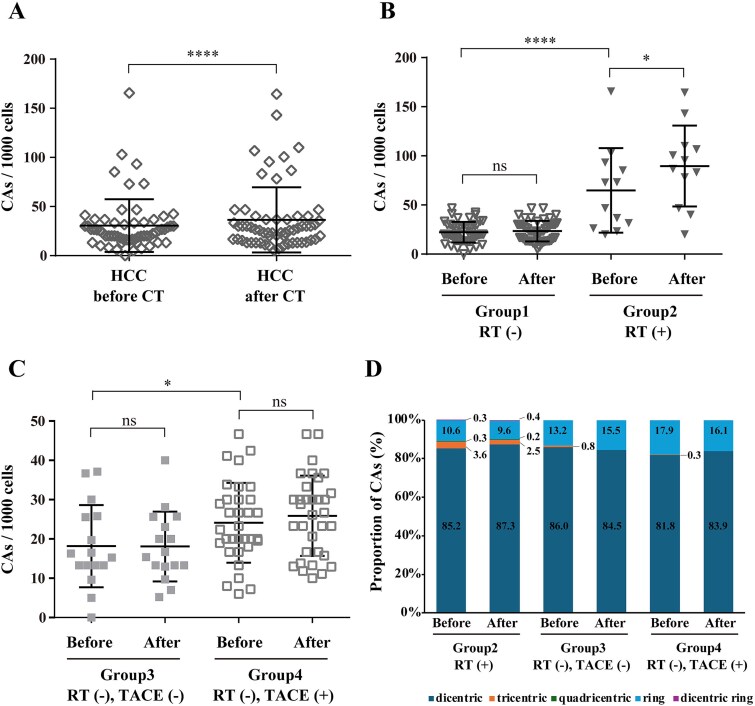
Subgroup analysis of HCC patients based on history of RT. (A) CAs in all 61 HCC patients before and after CT scans. The vertical axis indicates CAs per 1000 cells. (B) Changes in the first-level subgroup analysis (grouped by RT). (C) Changes in the second-level subgroup analysis (further grouped by TACE). The Wilcoxon signed-rank test was used to compare paired samples collected before and after a CT scan. Data are presented as mean ± SD. ^****^P < 0.0001; *P* < 0.05, ns: not significant. (D) Proportional distribution of CA types before and after CT in HCC patients (Groups 2–4). Stacked bars represent the percentage composition of each aberration type.

Among the 61 patients with HCC, the mean frequency of CAs per 1000 PBLs increased significantly after CT compared with before CT-scan levels (Fig. 3A; *P* < 0.0001), in line with previous reports. In a first-level subgroup analysis stratified by history of RT, patients without prior RT did not show a significant CT-related change in CAs, whereas those with a history of RT demonstrated a clear and significant increase (Fig. 3B, Supplementary Fig. S1A). Further grouping of the patients without prior RT subgroup, according to prior TACE revealed no appreciable difference in CT-induced CA changes between patients with and without TACE (Fig. 3C**,**  Supplementary Fig. S1B and C). Moreover, we also showed the distribution of unstable CAs (Fig. 3D). Complex CAs (including tricentric, quadricentric, and dicentric ring chromosomes) can be observed in patients with RT. These findings indicate that a history of RT exerts a strong influence on the biological response to CT exposure, whereas previous TACE has little impact on CA induction in this cohort.

### Individual variability in CAs response to CT

**Fig. 4:**
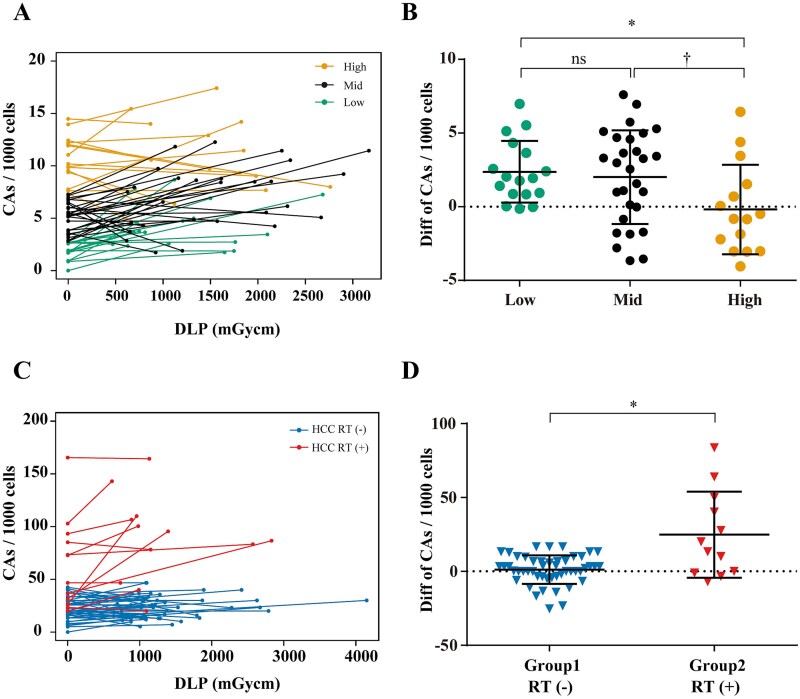
Individual differences in radiation sensitivity. (A) Subgroup analysis of 60 non-cancer patients before and after CT scans, stratified by DLP. Groups were defined based on the 25th and 75th percentiles of baseline CAs and classified as Low, Mid, and High. The vertical axis indicates the CAs per 1000 cells, horizontal axis indicates DLP (mGy^*^cm). (B) Differences in CAs for each group. The vertical axis shows the difference calculated as the value after minus the value before (CAs per 1000 cells). One-way analysis of variance (ANOVA) followed by Tukey’s honestly significant difference test was performed to evaluate statistical significance among groups. ^*^P < 0.05; † *P* < 0.10 (marginal significance); ns: not significant. (C) Subgroup analysis of 61 HCC patients before and after CT scans, stratified by DLP. (D) Differences in CAs for each group. Statistical significance was evaluated using the Mann–Whitney U test. ^****^P < 0.0001; ^*^P < 0.05.

In the non-cancer group, when patients were stratified into Low, Mid, and High categories based on their baseline CAs, a notable pattern emerged. Individuals in the low baseline CA group tended to show a greater increase in CAs after CT exposure, while those in the High baseline group exhibited a smaller increase or even a reduction in CA levels (Fig. 4A and B). This suggests that individuals with initially lower chromosomal damage may be more sensitive to radiation-induced changes.

In the HCC group, a comparison was made between patients with and without a history of RT. Those who had undergone RT showed a significantly larger increase in CAs after CT scans compared to those without RT history (Fig. 4C and D). This highlights a difference in radiation sensitivity related to prior radiation treatment.

Overall, our results illustrate individual variability in radiation response, with baseline CAs and prior RT history both influencing the magnitude of CA changes after CT.

### The correlation between CT-induced chromosome abnormalities and DLP

Multiple regression analysis revealed did not identify a direct association between DLP and the total **CA counts** (Tables 2 and 3, Supplementary Table S6). However, a previous study demonstrated a significant correlation between γH2AX foci formation and DLP even at low-dose CT exposure [35], reflecting direct DNA damage. Unlike γH2AX foci, which represent early DNA damage, CA formation is influenced by individual differences in DNA repair fidelity, thereby introducing substantial variability in CA measurements.

To evaluate the correlation between CAs and DLP, we constructed a simplified model assuming that CT-induced increase in CAs is proportional to DLP, with the regression coefficient dependent on the baseline CAs values (see Methods). To evaluate the effects of DLP on DIFF (After_CAs - Before_CAs), assuming the value of ‘Before_CAs’ to be average and fixing it at the mean, the equation can be rewritten as follows:


$$ DIFF=\left(a+b\cdot mean\ \left( Before\_ CAs\right)\right)\cdot DLP $$

**Fig. 5:**
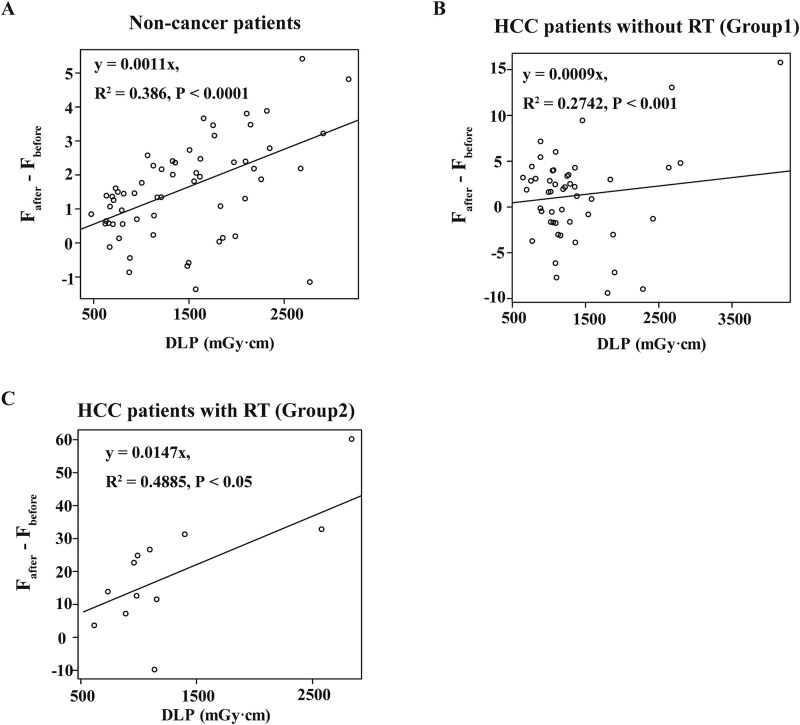
The formation of CAs after a CT scan positively correlated with DLP. The vertical axis indicates the fitted value of the CAs increased after a CT scan, calculated using the DLP value. Linear regression analysis was performed using data form several patient groups: (A) Non-cancer patients; (B) HCC patients without RT; (C) HCC patients with RT.

The effect of DLP on DIFF was examined using this transformed equation (Fig. 5).

Regression analysis of the non-cancer patients demonstrated a significant positive correlation between CT-induced CAs and DLP (R^2^ = 0.386, *P* < 0.0001) (Fig. 5A). A similar analysis of data from HCC patients demonstrated significant positive correlations between CT-induced CAs and DLP in both ‘HCC without RT’ (R^2^ = 0.2742, *P* < 0.001, Fig. 5B) and ‘HCC with RT’ (R^2^ = 0.4885, *P* < 0.05, Fig. 5C) groups. These findings indicate that CT-induced CAs increase with radiation dose, as reflected by DLP, after a CT scan.

## DISCUSSION

This is the first study to present a quantitative comparison of low-dose radiation responses between non-cancer patients and patients with HCC using the high-throughput PNA-FISH chromosome analysis approach. We demonstrated that baseline CAs in PBLs was substantially higher in HCC patients than in non-cancer individuals, with the greatest elevations observed in those with a history of RT (Fig. 2). We further found that HCC patients exhibited heterogeneous radiation responses to CT imaging and that CT-induced CAs were positively correlated with radiation dose. Importantly, the magnitude of CA induction was inversely related to the number of pre-existing CAs, indicating that individuals with lower baseline genomic damage showed greater sensitivity to low-dose irradiation.

A key finding of this study is that baseline CAs were significantly higher in HCC patients compared to non-cancer patients. Multivariate regression analysis identified the number of prior CT scans as a significant factor associated with baseline CAs in non-cancer patients (Supplementary Table S6). Among HCC patients, both TACE and RT histories showed strong associations with increased baseline CAs (Table 3). TACE involves the delivery of chemotherapeutic agents combined with embolization under fluoroscopic guidance, exposing patients to both radiation and chemotherapy. The radiation dose from TACE, assessed by total cumulative air kerma, has been reported to average 837.1 ± 571.0 (mGy) [42], which may contribute to the higher CAs observed in HCC patients. Although the direct genotoxic contribution of chemotherapeutic drugs used in TACE remains unclear, our findings suggest that both radiation and therapeutic interventions may compromise the genomic stability of peripheral lymphocytes in HCC patients. Collectively, these findings show that radiological diagnosis and treatment can compromise the chromosome stability of non-cancerous cells in patients.

**Fig. 6:**
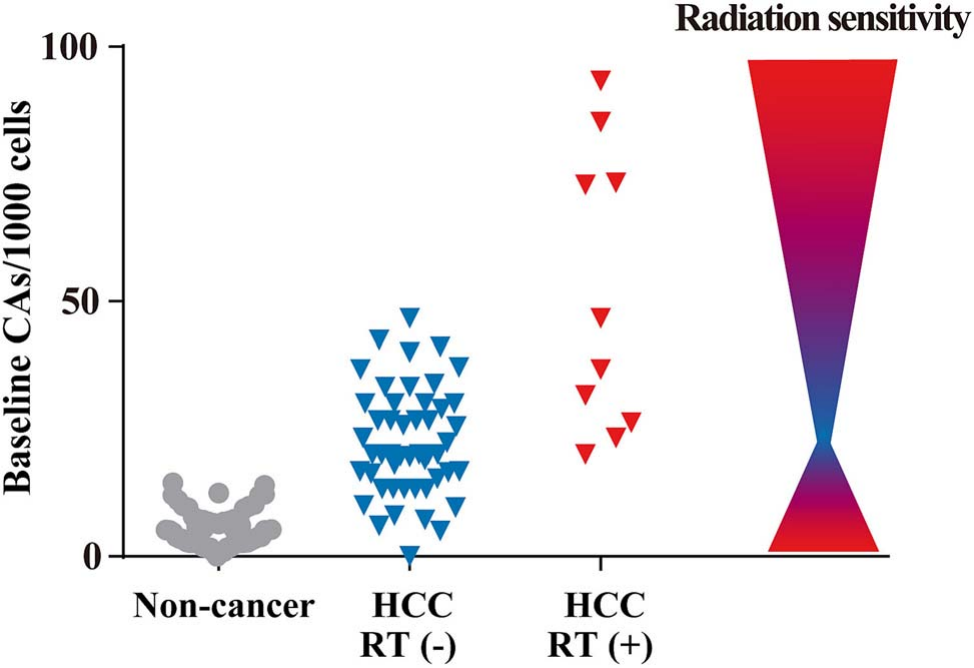
Graphical summary of the relationship between each group and radiation sensitivity. The vertical axis represents baseline CAs per 1000 cells. The heatmap on the right indicates the relative strength of radiation sensitivity.

Another notable observation is the differential CT-induced CA response among patient groups. Consistent with our earlier work in non-cancer patients, CAs increased significantly after CT scanning in many patients. Among these patients, those with lower baseline CAs exhibited a greater increase in CAs levels after CT scans. This inverse association suggests a baseline CA–dependent responsiveness to CT exposure; however, it does not necessarily indicate intrinsic radiosensitivity. Conversely, HCC patients without neither prior RT nor prior TACE showed no significant post-CT increase in CAs, despite having approximately fourfold higher baseline CAs than non-cancer individuals (Supplementary Fig. S2). The higher baseline CAs in HCC patients without RT could be attributed to DNA damage induced by repeated CT scans for follow-up, as well as various stressors associated with cancer [43, 44]. Such persistent low-level genomic damage may influence the apparent magnitude of CA induction after CT, rather than reflecting differences in intrinsic radiosensitivity. This mild, persistent DNA damage might have triggered a stronger adaptive response than in non-cancer patients, thereby reducing the increase in CAs after CT exposure. The observation that CT-induced increases in CAs were less apparent in patients with higher baseline levels does not contradict our previous findings [25]; rather, it represents a novel observation suggesting differential responsiveness associated with baseline status. In contrast, HCC patients with a history of RT showed a significant increase in CAs after CT scans, despite having the highest baseline CAs (Fig. 3B). This finding is consistent with the possibility that RT may alter DNA damage response or repair capacity, although alternative explanations cannot be excluded [45]. Importantly, while dicentrics constituted the majority of aberrations across all groups and no qualitative shift toward more complex aberrations was observed before and after CT exposure, complex aberrations such as tricentrics and quadricentrics were detected exclusively in patients with a history of radiotherapy (Fig. 3D). This finding suggests that these rare complex aberrations likely represent residual chromosomal damage from prior high-dose radiation exposure rather than newly induced effects of diagnostic CT. This baseline CA–dependent responsiveness to CT is illustrated as a conceptual model in Fig. 6. Further studies are needed to clarify the mechanisms of underlying adaptive response to low-dose radiation exposure.

One major limitation of this study is that baseline CA data were unavailable for HCC patients before enrollment, which prevented us from evaluating longitudinal CA trajectories or fully assessing the temporal persistence of genomic damage. Although age differed significantly between non-cancer and cancer groups, the average age of the cancer groups is inevitably higher than that of non-cancer patients; neither univariate nor multivariate analyses identified age as an independent predictor of CA levels. We also note that 11 of 12 HCC patients who received RT had a history of TACE, thereby complicating subgroup comparisons of these treatments. Nonetheless, the impact of RT correlated more strongly with baseline CAs than that of TACE (Table 3). Despite this overlap, our analysis suggests that RT strongly influences CA levels. Taken together, these results provide meaningful insights into the factors affecting baseline CAs among HCC patients. Future larger-scale studies with longitudinal designs are warranted to confirm our findings and more conclusively delineate the individual and combined effects of various treatment modalities on CA levels.

In this study, we demonstrated the importance of PNA-FISH analysis of PBLs as a potential tool for evaluating the radiation effects of CT scans. Although the cancer risk associated with CT scans was not directly assessed, we quantitatively measured individual differences in CT-induced chromosomal abnormalities, which may serve as markers of chromosomal radiation sensitivity. Our study suggests that standard CT scans increase CAs, which could contribute to cancer risk over time. Therefore, low-dose CT scans should be considered when they provide sufficient diagnostic efficacy. Additionally, cancer patients, who often have complex treatment histories and undergo frequent CT scans, tend to exhibit higher baseline CAs and increased sensitivity to low-dose irradiation. Given that cancer patients frequently undergo repeated imaging and complex therapies, careful justification and optimization of additional medical radiation exposures remain important.

Furthermore, guiding to avoid lifestyle factors—such as smoking—that may promote CAs represents a universally valuable recommendation for all patients. Developing high-throughput systems to analyse CAs could improve our understanding of the health effects of low-dose irradiation, optimize medical radiation protection strategies, and refine cancer therapy protocols.

## CONCLUSION

Our findings suggest that individuals with lower baseline numbers of abnormal chromosomes exhibit higher sensitivity to low-dose irradiation from CT scans. HCC patients had substantially higher baseline CA frequencies than non-cancer patients, especially those with complex treatment histories (notably prior RT). These observations underscore the need for careful consideration of cumulative medical radiation exposure, especially among cancer patients who undergo repeated imaging or treatment procedures.

## Supplementary Material

Supplementary_Matrials_rrag014
