# Supplementary material for: Radiation effects of a CT scan on chromosomal aberrations in cancer and non-cancer patients
Source: J Radiat Res. 2026 May 29;67(4):556–67. doi: 10.1093/jrr/rrag014 (PMC13400567; doi:10.1093/jrr/rrag014)
Supplement: Supplementary_Matrials_rrag014 [file supplementary_matrials_rrag014.zip › Supplementary Figures_ caption.docx]

**Supplementary Information**

**Supplementary Figure S1. Individual changes in CAs before and after CT in each subgroup**

Paired plots show CA frequencies (per 1000 cells) for each individual before and after CT in (A) Group 2 (RT+), (B) Group 3 (RT−, TACE−), and (C) Group 4 (RT−, TACE+). Each line represents an individual participant. Statistical comparisons between before and after-CT values were performed using the Wilcoxon signed-rank test. *P* < 0.05, ns indicates not significant.

**Supplementary Figure S2. Chromosome aberrations in PBLs of non-cancer and HCC patients without RT nor TACE before and after CT scans**

Comparisons before and after CT scans within the same group were conducted using the Wilcoxon signed-rank test. The Mann-Whitney U test was used to assess significance between different groups. Data represent means ± SD. **** *P* < 0.0001, * *P* < 0.05.
