# Supplementary material for: Radiation effects of a CT scan on chromosomal aberrations in cancer and non-cancer patients
Source: J Radiat Res. 2026 May 29;67(4):556–67. doi: 10.1093/jrr/rrag014 (PMC13400567; doi:10.1093/jrr/rrag014)
Supplement: Supplementary_Matrials_rrag014 [file supplementary_matrials_rrag014.zip › Supplementary Tables_v4.docx]

**Supplementary Table S1. Number of CAs per 1,000 PBLs in all patients before and after CT scans**

| ID | History of TACE | History of RT | Gender | Age | DLP | Effective dose | Contrast agent | Before CT | | After CT | |
| --- | --- | --- | --- | --- | --- | --- | --- | --- | --- | --- | --- |
|  | (Yes, No) | (Yes, No) | (m,f) | (years) | (mGy･cm) | (mSv) |  | Total cells | CAs/1,000 cells | Total cells | CAs/1,000 cells |
| 1 | Yes | Yes | m | 69 | 615 | 8.6 | Iopamidol | 1146 | 118 | 1090 | 156 |
| 2 | Yes | Yes | m | 58 | 1134 | 15.9 | Iomeprol | 302 | 50 | 304 | 50 |
| 3 | Yes | No | m | 69 | 4153 | 58.1 | Iomeprol | 300 | 4 | 300 | 4 |
| 4 | No | No | m | 73 | 877 | 12.3 | Unknown | 313 | 8 | 312 | 8 |
| 5 | Yes | No | m | 78 | 1087 | 15.2 | Unknown | 1122 | 9 | 1179 | 14 |
| 6 | Yes | Yes | m | 61 | 1395 | 19.5 | Iomeprol | 316 | 10 | 314 | 30 |
| 7 | No | No | f | 66 | 1171 | 16.4 | Iohexol | 310 | 8 | 311 | 8 |
| 8 | Yes | Yes | m | 84 | 980 | 13.7 | Unknown | 453 | 33 | 199 | 20 |
| 9 | Yes | Yes | m | 74 | 2832 | 39.6 | Iopamidol | 300 | 11 | 300 | 26 |
| 10 | Yes | No | m | 85 | 689 | 9.6 | Iopamidol | 300 | 5 | 300 | 8 |
| 11 | No | No | m | 54 | 879 | 12.3 | Iomeprol | 300 | 0 | 308 | 3 |
| 12 | Yes | No | m | 74 | 1019 | 14.3 | Iohexol | 300 | 6 | 300 | 7 |
| 13 | Yes | Yes | f | 79 | 957 | 13.4 | Iomeprol | 304 | 8 | 300 | 33 |
| 14 | Yes | No | m | 68 | 1832 | 25.6 | Iomeprol | 300 | 6 | 300 | 4 |
| 15 | Yes | No | m | 62 | 879 | 12.3 | Iopamidol | 1000 | 6 | 1000 | 13 |
| 16 | No | No | m | 44 | 1350 | 18.9 | Iomeprol | 1178 | 18 | 1032 | 29 |
| 17 | Yes | No | m | 67 | 1088 | 15.2 | Iomeprol | 300 | 9 | 300 | 14 |
| 18 | Yes | No | m | 62 | 1280 | 17.9 | Iomeprol | 1174 | 34 | 1179 | 42 |
| 19 | Yes | No | m | 69 | 2671 | 37.4 | Unknown | 300 | 3 | 300 | 7 |
| 20 | Yes | Yes | m | 63 | 1093 | 15.3 | Iomeprol | 300 | 7 | 300 | 6 |
| 21 | No | No | m | 93 | 1890 | 26.5 | Iohexol | 300 | 11 | 300 | 12 |
| 22 | Yes | No | m | 82 | 893 | 12.5 | Iopamidol | 300 | 8 | 300 | 11 |
| 23 | No | No | m | 77 | 1018 | 14.3 | Iopamidol | 300 | 9 | 300 | 4 |
| 24 | Yes | No | m | 68 | 1188 | 16.6 | Iomeprol | 300 | 6 | 300 | 7 |
| 25 | Yes | Yes | f | 82 | 988 | 13.8 | Iomeprol | 300 | 6 | 300 | 12 |
| 26 | Yes | Yes | m | 68 | 2573 | 36.0 | Iohexol | 300 | 22 | 300 | 25 |
| 27 | No | No | f | 65 | 749 | 10.5 | Iohexol | 300 | 4 | 300 | 6 |
| 28 | No | No | m | 58 | 1044 | 14.6 | Unknown | 300 | 4 | 300 | 4 |
| 29 | Yes | No | m | 72 | 1532 | 21.4 | Iomeprol | 300 | 8 | 300 | 7 |
| 30 | Yes | No | m | 64 | 989 | 13.8 | Iohexol | 300 | 6 | 300 | 11 |
| 31 | Yes | No | m | 71 | 1033 | 14.5 | Iopamidol | 300 | 8 | 300 | 9 |
| 32 | Yes | No | m | 67 | 1119 | 15.7 | Iohexol | 300 | 10 | 300 | 10 |
| 33 | Yes | No | m | 63 | 2415 | 33.8 | Iomeprol | 300 | 8 | 300 | 12 |
| 34 | No | Yes | f | 75 | 732 | 10.3 | Unknown | 300 | 14 | 300 | 14 |
| 35 | Yes | No | f | 66 | 1152 | 16.1 | Iopamidol | 300 | 10 | 300 | 11 |
| 36 | No | No | m | 66 | 813 | 11.4 | Iopamidol | 300 | 4 | 300 | 4 |
| 37 | Yes | No | f | 79 | 1052 | 14.7 | Iohexol | 300 | 9 | 300 | 9 |
| 38 | Yes | Yes | f | 62 | 886 | 12.4 | Iohexol | 300 | 28 | 300 | 32 |
| 39 | Yes | No | m | 70 | 1870 | 26.2 | Iohexol | 300 | 9 | 300 | 9 |
| 40 | Yes | Yes | f | 78 | 1152 | 16.1 | Iopamidol | 1127 | 96 | 1163 | 91 |
| 41 | Yes | No | m | 65 | 1214 | 17.0 | Iomeprol | 307 | 6 | 306 | 8 |
| 42 | No | No | m | 69 | 640 | 9.0 | Iomeprol | 1141 | 11 | 1105 | 17 |
| 43 | No | No | f | 50 | 1056 | 14.8 | Iomeprol | 300 | 4 | 300 | 5 |
| 44 | No | No | m | 55 | 1006 | 14.1 | Iohexol | 1041 | 17 | 763 | 4 |
| 45 | Yes | No | m | 74 | 1375 | 19.3 | Iohexol | 1116 | 25 | 1114 | 23 |
| 46 | Yes | No | m | 70 | 1794 | 25.1 | Unknown | 316 | 13 | 317 | 5 |
| 47 | Yes | No | m | 78 | 1283 | 18.0 | Iohexol | 1106 | 21 | 1083 | 14 |
| 48 | Yes | No | f | 74 | 761 | 10.7 | Unknown | 1109 | 8 | 1081 | 12 |
| 49 | Yes | No | m | 70 | 1346 | 18.8 | Iomeprol | 300 | 6 | 300 | 5 |
| 50 | Yes | No | f | 75 | 766 | 10.7 | Iohexol | 300 | 12 | 300 | 5 |
| 51 | Yes | No | f | 68 | 1097 | 15.4 | Iopamidol | 300 | 14 | 300 | 14 |
| 52 | No | No | m | 69 | 1262 | 17.7 | Iohexol | 303 | 5 | 308 | 4 |
| 53 | Yes | No | m | 57 | 2630 | 36.8 | Iohexol | 300 | 6 | 300 | 9 |
| 54 | Yes | No | f | 66 | 1243 | 17.4 | Unknown | 300 | 5 | 300 | 9 |
| 55 | No | No | m | 58 | 2790 | 39.1 | Iomeprol | 304 | 6 | 300 | 6 |
| 56 | Yes | No | m | 77 | 1080 | 15.1 | Iomeprol | 388 | 7 | 435 | 6 |
| 57 | Yes | No | m | 60 | 1354 | 19.0 | Iohexol | 325 | 11 | 312 | 9 |
| 58 | No | No | m | 64 | 2277 | 31.9 | Iomeprol | 323 | 12 | 347 | 8 |
| 59 | Yes | No | m | 77 | 1576 | 22.1 | Iomeprol | 300 | 7 | 300 | 3 |
| 60 | Yes | No | m | 68 | 1083 | 15.2 | Iohexol | 306 | 13 | 316 | 10 |
| 61 | No | No | m | 80 | 1451 | 20.3 | Iomeprol | 1000 | 5 | 1000 | 7 |

The counts of CAs are the raw count without normalization.

**Supplementary Table S2. Mean incidence of CAs per 1,000 PBLs in HCC patients before CT (related to Fig. 2A)**

|  | Before CT |
| --- | --- |
| Non-cancer all, (n=60) | 5.6 ± 3.6 |
| HCC all, (n=61) | 30.6 ± 26.8 |

Data are shown as mean ± SD.

**Supplementary Table S3. Radiotherapy history and interval between radiotherapy and enrollment in the CT study for 12 HCC patients**

| Patient ID | Dose (Gy)/Number of fractions | Period between RT and enrollment in the CT study (months) |
| --- | --- | --- |
| 1 | 48 Gy/4 | 9 |
| 5 | 48 Gy/4 (1st RT);  48 Gy/4 (2nd RT) | 42 (1st RT~);  35 (2nd RT~) |
| 13 | 48 Gy/4 | 34 |
| 16 | 45 Gy/15 | 63 |
| 17 | Heavy ion radiotherapy;  no detail | 11 |
| 22 | 48 Gy/4 | 42 |
| 31 | 48 Gy/4 | 36 |
| 37 | 45 Gy/4 | 91 |
| 39 | 39 Gy/4 | 32 |
| 58 | 40 Gy/4 | 25 |
| 64 | 60 Gy/4 | 24 |
| 66 | 48 Gy/4 | 27 |
|  |  |  |

**Supplementary Table S4. Mean incidence of CAs per 1,000 PBLs in HCC patients before and after CT scans (related to Fig. 2A, B)**

|  | Before CT | After CT |
| --- | --- | --- |
| HCC all, (n=61) | 30.6 ± 26.8 | 36.4 ± 33.2 |
| HCC RT (-), (n=49) | 22.2 ±10.5 | 23.4 ± 10.4 |
| HCC RT (+), (n=12) | 64.8 ± 43.1 | 89.6 ± 41.2 |

Data are shown as mean ± SD

**Supplementary Table S5. Mean incidence of CAs per 1,000 PBLs in HCC patients without RT before and after CT scans (related to Fig. 2C)**

|  | Before CT | After CT |
| --- | --- | --- |
| HCC RT (-) all, (n=49) | 22.2 ± 10.5 | 23.4 ± 10.4 |
| HCC RT (-) TACE (-), (n=16) | 18.2 ± 10.5 | 18.1 ± 8.9 |
| HCC RT (-) TACE (+), (n=33) | 24.1 ± 10.2 | 25.9 ± 10.2 |

Data are shown as mean ± SD

**Supplementary Table S6.** **Univariate and multiple regression analysis of factors affecting the number of CAs before CT scans among non-cancer patients**

|  | Univariate test | |  | | Multivariate test | | |  | |  |
| --- | --- | --- | --- | --- | --- | --- | --- | --- | --- | --- |
| Variable | Coefficient | p-value | |  | | Coefficient | p-value | |  | |
| Sex | 1.770 | 0.323 | |  | |  |  | |  | |
| Age | 0.030 | 0.672 | |  | |  |  | |  | |
| BMI | 0.147 | 0.621 | |  | |  |  | |  | |
| DLP | 1.136×10^-3^ | 0.459 | |  | |  |  | |  | |
| History of smoking | 0.221 | 0.903 | |  | | -1.087 | 0.526 | |  | |
| Number of CT scans | 1.059 | 3.179×10^-2^ | | * | | 1.163 | 2.981×10^-2^ | | * | |

* p < 0.05
