# Supplementary figures and images for: Radiation effects of a CT scan on chromosomal aberrations in cancer and non-cancer patients
Source: J Radiat Res. 2026 May 29;67(4):556–67. doi: 10.1093/jrr/rrag014 (PMC13400567; doi:10.1093/jrr/rrag014)

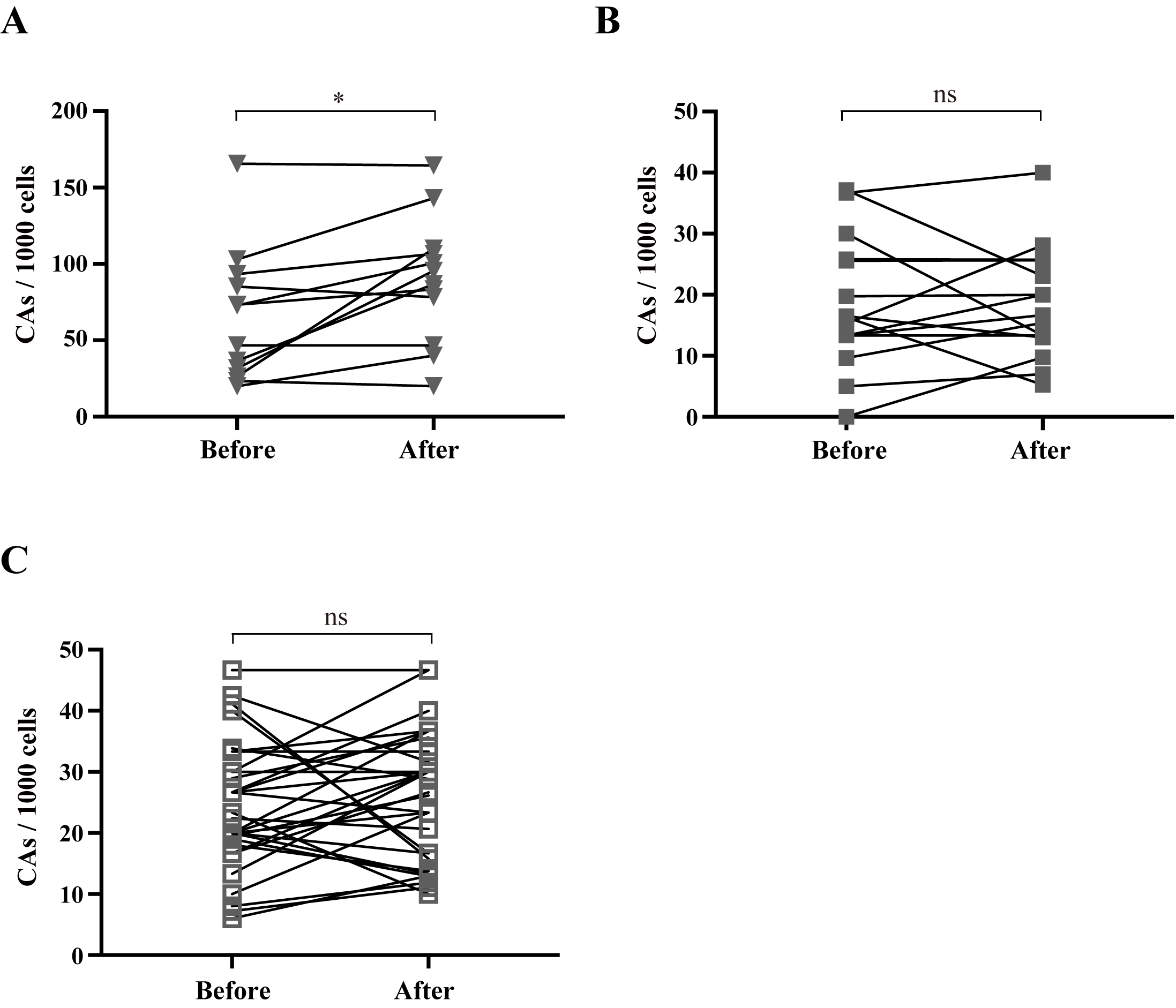

Supplement: Supplementary_Matrials_rrag014 [file supplementary_matrials_rrag014.zip › Supplementary_Figure_S1_individualplots_v0.tif]

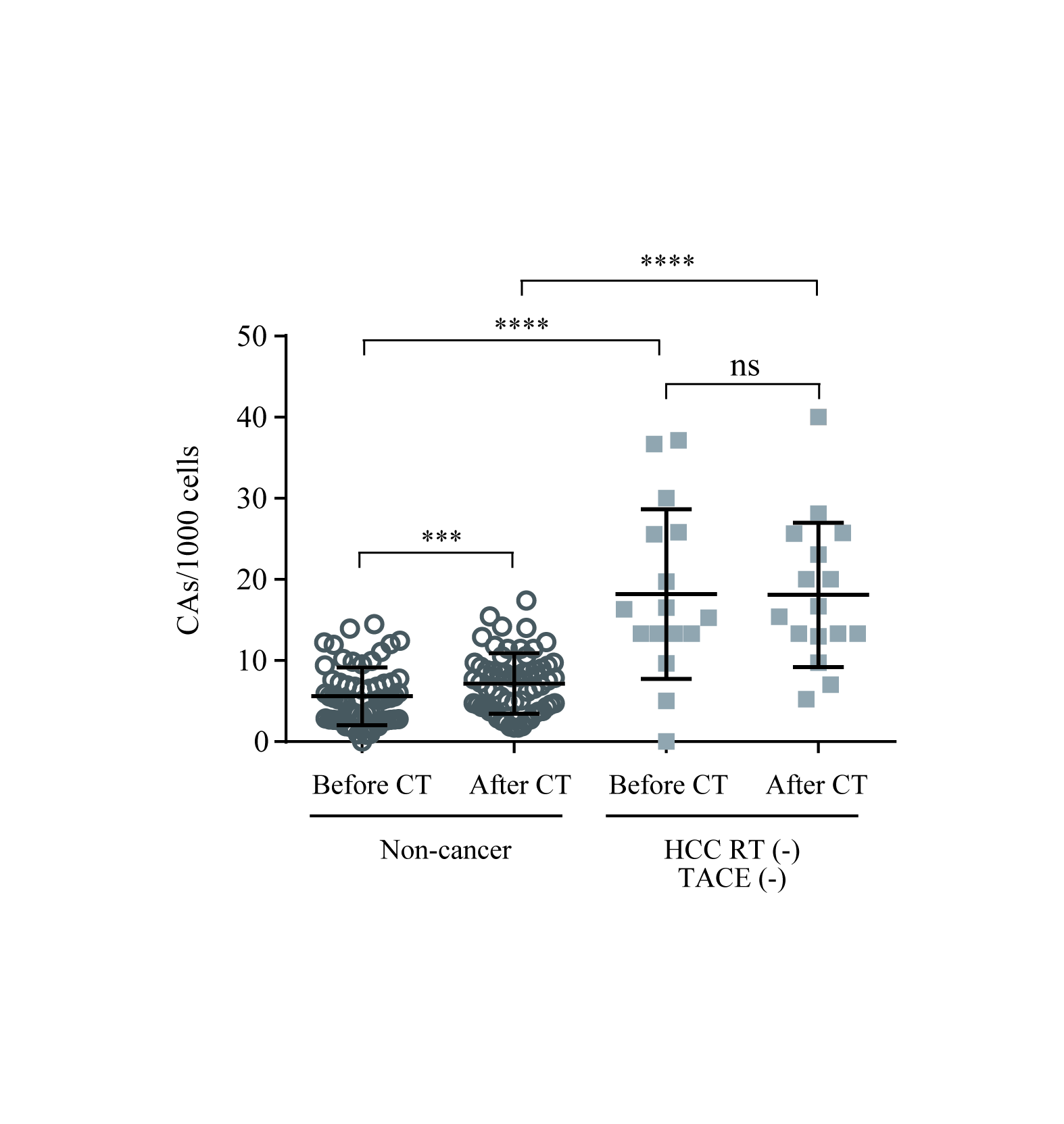

Supplement: Supplementary_Matrials_rrag014 [file supplementary_matrials_rrag014.zip › Supplementary_fig_S2_v1.tif]
